# Transfer of microRNA-22-3p by M2 macrophage-derived extracellular vesicles facilitates the development of ankylosing spondylitis through the PER2-mediated Wnt/β-catenin axis

**DOI:** 10.1038/s41420-022-00900-1

**Published:** 2022-05-23

**Authors:** Chong Liu, Tuo Liang, Zide Zhang, Jiarui Chen, Jang Xue, Xinli Zhan, Liang Ren

**Affiliations:** 1 Spine and Osteopathy Ward, The First Affiliated Hospital of Guangxi Medical University, Nanning, 530021 P. R. China; 2 Guangxi Medical University, Nanning, 530021 P. R. China; 3 Reproductive Medicine Center, The First Affiliated Hospital of Guangxi Medical University, Nanning, 530021 P.R. China

**Keywords:** Rheumatic diseases, Cell biology

## Abstract

Pathological osteogenesis and inflammation possess critical significance in ankylosing spondylitis (AS). The current study aimed to elucidate the mechanisms regarding extracellular vesicle (EV)-packaged microRNA-22-3p (miR-22-3p) from M2 macrophages in the osteogenic differentiation of mesenchymal stem cells (MSCs) in AS. EVs were initially isolated from M2 macrophages, which had been treated with either restored or depleted miR-22-3p. AS-BMSCs were subsequently treated with M2 macrophage-derived EVs to detect osteogenic differentiation in BMSCs using gain- or loss-of-function experiments. The binding affinity among miR-22-3p, period circadian protein 2 (PER2), and Wnt7b was identified. Finally, AS mouse models were established for testing the effects of M2-EV-miR-22-3p on the bone metastatic microenvironment in vivo. miR-22-3p from M2 macrophages could be transferred into BMSCs via EVs, which promoted the osteogenic differentiation of AS-BMSCs. miR-22-3p inhibited PER2, while PER2 blocked the Wnt/β-catenin signaling pathway via Wnt7b inhibition. M2-EV-shuttled miR-22-3p facilitated alkaline phosphatase activity and extracellular matrix mineralization via PER2-regulated Wnt/β-catenin axis, stimulating the BMSC osteogenic differentiation. Taken together, these findings demonstrate that miR-22-3p in M2 macrophage-released EVs downregulates PER2 to facilitate the osteogenesis of MSCs via Wnt/β-catenin axis.

## Introduction

As a chronic autoimmune disorder, ankylosing spondylitis (AS) is characterized by inflammatory back pain during the early stages of the disease, which often results in movement restriction as the disease progresses, and eventually complete disability [1]. AS has been well documented to result in high rates of spinal fractures owing to the event of early-onset osteoporosis [2]. The mechanism underpinning the pathological osteogenesis of AS continues to be researched [3, 4]. Liu et al. asserted that mesenchymal stem cells (MSCs) from AS sufferers contribute to the pathological osteogenesis observed in AS [5]. MSCs are crucial pluripotent stem cells that possess significant immune-regulatory properties as well as three lineage-differentiation abilities [6]. However, the specific mechanisms underlying the inflammation observed in the event of pathological osteogenesis in AS remain largely unknown [7].

Extracellular vesicles (EVs) can be released by various kinds of cells, regardless of normal and pathological conditions, including hepatocytes [8]. Serum-derived EVs have been well documented to influence the progression of AS [9]. Macrophages represent the chief components of tumor-infiltrating immune cells, with M2 macrophage-derived EVs (M2-EVs) linked by a previous report to tumor progression [10]. The significant role of EVs in the process of cell-to-cell communication in the setting of both physiological and pathological events has been highlighted, as it can act as a carrier of various biomolecules, including microRNAs (miRNAs) [11]. Specifically, miR-22-3p is capable of regulating various autoimmune diseases [12]. Aberrant expression of miR-22-3p in AS has been previously reported [13]. During the current study, the TargetScan website provided data verifying that miR-22-3p could target period circadian protein 2 (PER2). As central regulators of the metazoan circadian (daily) clock, PERIOD (PER) proteins have been identified in Drosophila as a gene whose mutation results in a short or long period, creating the modern era of molecular circadian biology [14]. Depleted PER2 has been implicated to impair normal behavior and circadian rhythm, which also significantly increases tumor incidence and proliferation of abnormal phenotypes [15]. Besides, PER2 possesses potential function in influencing the proliferation and stemness of glioma stem cells in relation to the Wnt/β-catenin signaling pathway to influence [16]. However, the mechanism by which M2-EV communication influences osteogenic differentiation in AS in connection with the interplay between miR-22-3p, PER2, and Wnt/β-catenin, is still poorly understood, highlighting a major gap in knowledge, given that M2-EVs may be of significance in the process of osteogenic differentiation in AS. Hence, we asserted the hypothesis that the transfer of miR-22-3p via M2-EVs could potentially alter the osteogenesis in AS.

## Results

### M2-EVs triggered the osteogenic differentiation of AS-BMSCs

**Fig. 1:**
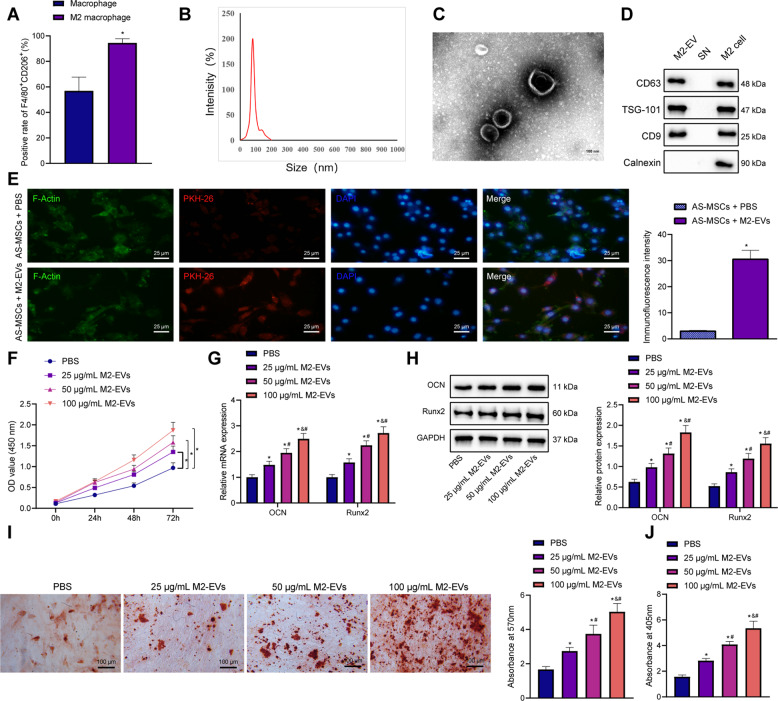
Effects of M2-EVs on the viability and osteogenic differentiation of AS-BMSCs. **A** The expression of M2 macrophage markers detected by flow cytometry. **B** The particle size of M2-EVs analyzed by DLS. **C** Ultrastructure of M2-EVs observed by a TEM (scale bar = 100 nm). **D** The expression of EV-marker proteins in the M2-EVs measured using Western blot analysis. SN, supernatant. **E** The uptake of M2-EVs labeled with fluorescence PKH26 by the AS-BMSCs observed under an immunofluorescence microscope (scale bar = 25 μm). PKH26-labeled EVs were red, DAPI-stained nuclei were blue, and F-actin-labeled cytoskeleton was green. **F** AS-BMSC viability following coculture with M2-EVs at different concentrations at different time points detected by CCK-8. **G** The mRNA expression of Runx2 and OCN, osteogenic differentiation markers in AS-BMSCs cocultured with M2-EVs at different concentrations for 7 days, determined by RT-qPCR. **H** The protein expression of Runx2 and OCN, osteogenic differentiation markers in AS-BMSCs cocultured with M2-EVs at different concentrations for 7 days, determined by Western blot analysis. **I** The mineralization capacity of AS-BMSCs cocultured with M2-EVs at different concentrations for 14 days detected using alizarin red staining, scale bar = 100 μm. **J** Osteogenic differentiation of AS-BMSCs cocultured with M2-EVs at different concentrations for 14 days detected by ALP staining. *p < 0.05 vs. M2 macrophages/AS-BMSCs + PBS. #*p* < 0.05 vs. AS-BMSCs treated with 25 μg/mL M2-EVs/THP-1-EVs /M2-EVs + inhibitor NC, & *p* < 0.05 vs. AS-BMSCs treated with 50 μg/mL M2-EVs. The cell experiment was repeated three times independently.

To unfold the effect of M2-EVs on the osteogenic differentiation of AS-BMSCs, we initially stimulated THP-1 cells to differentiate into primary macrophages by PMA. Microscopic observation results showed that the cell morphology changed significantly, the ratio of cytoplasm was increased, and the cell adherence was better after differentiation into macrophages. The undifferentiated THP-1 cells were then removed from the culture dish using the adherence characteristics (Figure S1). Next, the macrophages were incubated with IL-4 for 72 h to induce polarization to M2 phenotype. In addition, F4/80 and CD206 double-positive flow cytometry was used to isolate M2 macrophages. The results showed an increase in the ratio of M2 macrophages (Fig. 1A), indicating that the purity of M2 macrophages was very high after isolation.

Next, EVs were extracted from M2 macrophages. DLS results displayed that there were particles with the size of 30 and 200 nm in the sample (Fig. 1B). TEM observation indicated that these particles had typical bilayer-membrane structure and were cup-shaped or spherical (Fig. 1C). Besides, EV-marker proteins CD63, CD81, and TSG-101 were positive, while calnexin was negative (Fig. 1D). The aforementioned results suggested the successful isolation of EVs.

We subsequently set out to ascertain whether M2-EVs could be internalized by AS-BMSCs. PKH26-labeled M2-EVs were cocultured with AS-BMSCs for 12 h and obvious red fluorescence could be observed in the AS-BMSCs (Fig. 1E), indicating the uptake of M2-EVs by the AS-BMSCs. Varying doses of M2-EVs were cocultured with AS-BMSCs. Cell counting kit-8 (CCK-8) assay results (Fig. 1F) revealed that following coculture with M2-EVs, the activity of AS-BMSCs increased in a dose-dependent manner. To further evaluate the effect of M2-EVs on the osteogenesis of AS-BMSCs, the expression of osteogenic differentiation markers Runx2 and osteocalcin (OCN) was detected by reverse-transcription quantitative polymerase-chain reaction (RT-qPCR) and Western blot analysis in AS-BMSCs after coculture in OM containing M2-EVs at different concentrations for 7 days. The results obtained revealed increases in Runx2 and OCN in AS-BMSCs post coculture with M2-EVs, showing a dose-dependent manner (Fig. 1G, H). After coculture with M2-EVs at different concentrations for 14 days, AS-BMSCs were subjected to alizarin red staining and alkaline phosphatase (ALP) staining. The results demonstrated that the mineralization ability and ALP content of the AS-BMSCs cocultured with M2-EVs were increased dose dependently (Fig. 1I, J).

### M2-EVs carrying miR-22-3p induced the osteogenic differentiation of AS-BMSCs

**Fig. 2:**
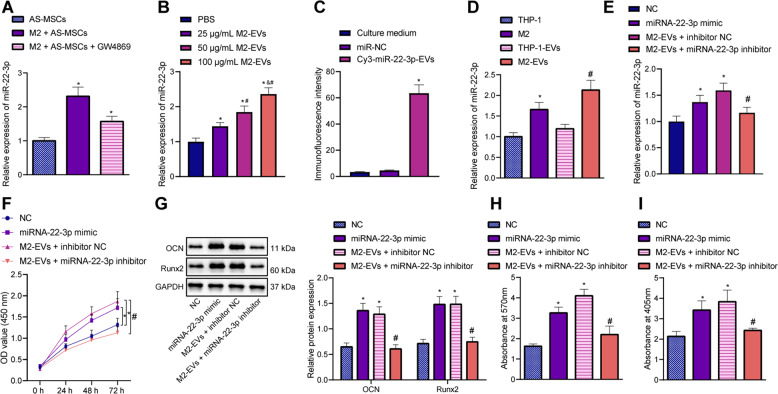
Effects of M2-EVs carrying miR-22-3p on the osteogenic differentiation of AS-BMSCs. **A** miR-22-3p expression determined by RT-qPCR in AS-BMSCs cocultured with M2-EVs or those treated with 5 μM GW4869. **B** miR-22-3p expression determined by RT-qPCR in AS-BMSCs cocultured with M2-EVs at different concentrations. **C** Uptake of M2-EVs carrying Cy3–miR-22-3p by AS-BMSCs observed under a fluorescence microscope. Cy3-labeled miR-22-3p was red, DAPI-stained nuclei were blue, and phalloidin-labeled cytoskeleton was green. **D** miR-22-3p expression determined by RT-qPCR in THP-1 cells, M2 macrophages, M2-EVs, and THP-1-EVs. **E** miR-22-3p expression in AS-BMSCs treated with M2-EVs and/or miR-22-3p mimic/inhibitor determined by RT-qPCR. **F** The viability of AS-BMSCs treated with M2-EVs and/or miR-22-3p mimic/inhibitor detected by CCK-8 assay. **G** The protein expression of Runx2 and OCN in AS-BMSCs treated with M2-EVs and/or miR-22-3p mimic/inhibitor measured using Western blot analysis. **H** Mineralization of AS-BMSCs treated with M2-EVs and/or miR-22-3p mimic/inhibitor detected using Alizarin red staining. **I** ALP content of AS-BMSCs treated with M2-EVs and/or miR-22-3p mimic/inhibitor detected by ALP staining. *p < 0.05 vs. AS-BMSCs treated with PBS/THP-1/NC, #*p* < 0.05 vs. AS-BMSCs treated with 25 μg/mL M2-EVs/THP-1-EVs /M2-EVs + inhibitor NC, and *p* < 0.05 vs. AS-BMSCs treated with 50 μg/mL M2-EVs. The cell experiment was repeated three times independently.

We found an elevation in miR-22-3p expression in AS-BMSCs cocultured with M2 macrophages, while a reduction after GW4869 (Fig. 2A). In addition, M2-EVs caused an enhancement in miR-22-3p in the AS-BMSCs, presenting with a dose-dependent manner (Fig. 2B). Moreover, treatment of M2-EVs with RNase A and Proteinase K did not affect miR-22-3p expression in AS-BMSCs increasing with the concentration of M2-EVs, but treatment with Triton X-100 led to no changes in miR-22-3p expression in AS-BMSCs (Figure S2). Next, M2 macrophages were transfected with Cy3-labeled miR-22-3p and M2-EVs were cocultured with AS-BMSCs. The Cy3-labeled M2-EVs–miR-22-3p was predominately located in the cytoplasm of AS-BMSCs (Fig. 2C). The above results indicated that M2-EVs can significantly increase miR-22-3p expression in AS-BMSCs, and miR-22-3p was carried by M2-EVs into AS-BMSCs. In order to make miR-22-3p be more enriched in AS-BMSCs, we subsequently used 100 μg/mL M2-EVs to treat AS-BMSCs. We discovered that miR-22-3p was highly expressed in M2 macrophages and M2-EVs (Fig. 2D).

In an attempt to ascertain whether M2-EVs could transfer miR-22-3p to AS-BMSCs and promote the osteogenic differentiation of AS-BMSCs, AS-BMSCs were transfected with gain/loss-of-function of miR-22-3p or cocultured with M2-EVs. We observed that AS-BMSCs treated with gain-of-function of miR-22-3p or M2-EVs exhibited an increase in miR-22-3p expression, heightened cell viability, in addition to elevated levels of Runx2 and OCN, with opposite results observed in the AS-BMSCs treated with loss-of-function of miR-22-3p following coculture with M2-EVs (Fig. 2E–G). Furthermore, observation revealed that cell mineralization and ALP content were enhanced in AS-BMSCs treated with gain-of-function of miR-22-3p or M2-EVs, while contrary results were noted in AS-BMSCs cocultured with M2-EVs and treated with loss-of-function of miR-22-3p (Fig. 2H, I). These data suggested that miR-22-3p shuttled by M2-EVs contributed to the osteogenic differentiation of AS-BMSCs.

### miR-22-3p carried by M2-EVs targeted PER2 and inhibited the expression of PER2

**Fig. 3:**
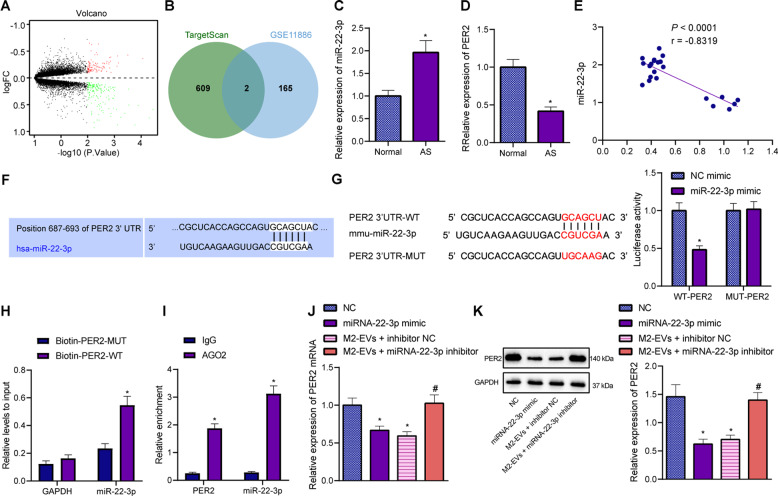
The targeting relationship between miR-22-3p and PER2. **A** A volcano map of significantly differentially expressed mRNAs in normal samples (*n* = 9) and AS samples (*n* = 8) in the AS-related dataset GSE11886. **B** Venn diagram of potential target genes of miR-22-3p. The green circle represents the target genes predicted by TargetScan database, and the blue circle represents the downregulated genes in the GSE11886 dataset (9 normal samples and 8 AS samples). **C** Expression of miR-22-3p in normal spinal ligaments of patients with spinal fractures (*n* = 6) and spinal ligaments of patients with AS (*n* = 16) determined by RT-qPCR. **D** The mRNA expression of PER2 in the normal spinal ligaments of patients with spinal fractures (*n* = 6) and spinal ligaments of patients with AS (*n* = 16) determined by RT-qPCR. **E** The correlation between miR-22-3p expression and PER2 expression in the spinal ligaments of patients with AS (*n* = 16) analyzed by Pearson’s correlation coefficient. **F** The binding sites between miR-22-3p and PER2 predicted by TargetScan. **G** The target relationship between miR-22-3p and PER2 verified by dual-luciferase reporter gene assay. **H** Interaction between miR-22-3p and PER2 verified by RNA pull-down assay. **I** Enrichment of miR-22-3p and PER2 in cells incubated with AGO2 measured by RIP assay. **J** The mRNA expression of PER2 in the AS-MSCs treated with M2-EVs and/or miR-22-3p mimic/inhibitor measured by RT-qPCR. **K** The protein expression of PER2 in the AS-BMSCs treated with M2-EVs and/or miR-22-3p mimic/inhibitor measured by Western blot analysis. *p < 0.05 vs. normal spinal ligaments/biotin–PER2-WT/IgG/NC, #*p* < 0.05 vs. AS-BMSCs treated with M2-EVs + inhibitor NC. The cell experiment was repeated three times independently.

Next, to further elucidate the mechanism of miR-22-3p in AS-BMSCs, the target genes of miR-22-3p were predicted by TargetScan database. Differential-analysis results yielded 106 significantly upregulated mRNAs and 167 significantly downregulated mRNAs in the samples of patients with AS (Fig. 3A). The target genes of miR-22-3p were intersected with the downregulated genes in the AS samples in the GSE11886 dataset, and the results unfolded that BTG1 and PER2 were the potential target genes of miR-22-3p (Fig. 3B), in which the inhibition of PER2 has been demonstrated to exert a stimulatory effect on the osteogenic differentiation of MSCs [22]. Therefore, PER2 was selected as the research object.

It was evident that miR-22-3p was obviously elevated (Fig. 3C) and PER2 was reduced in the spinal ligaments of patients with AS (Fig. 3D). Further, Pearson’s correlation coefficient (Fig. 3E) analyzed a negative association between miR-22-3p and PER2 in the spinal ligaments of patients with AS. The potential binding site of miR-22-3p with PER2 3′ untranslated region was predicted by TargetScan website (Fig. 3F), which was further validated by luciferase assay that the luciferase activity of PER2 wild type (WT) was inhibited by miR-22-3p mimic, with no significant difference detected in PER2 mutant (MUT) (Fig. 3G). Furthermore, RNA pull-down assay results showed a direct interaction between miR-22-3p and PER2 mRNA (Fig. 3H), and RIP experimental results revealed that AGO2 could simultaneously enrich miR-22-3p and PER2 mRNA (Fig. 3I). The expression of PER2 was decreased in AS-BMSCs treated with gain-of-function of miR-22-3p or M2-EVs, while it was elevated in AS-BMSCs treated with loss-of-function of miR-22-3p and M2-EVs (Fig. 3J, K). Thus, miR-22-3p encapsulated by M2-EVs limited PER2 expression.

### miR-22-3p promoted the osteogenic differentiation of AS-BMSCs by inhibiting PER2

**Fig. 4:**
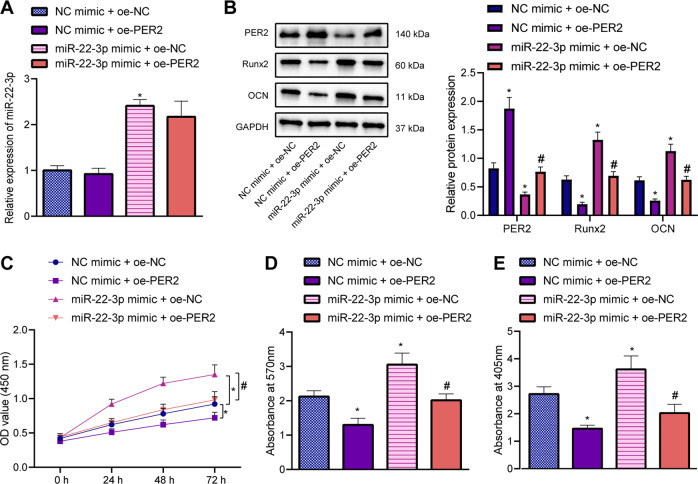
miR-22-3p repressed PER2 to induce the osteogenic differentiation of AS-BMSCs. **A** miR-22-3p expression in AS-BMSCs treated with miR-22-3p mimic, oe-PER2, or in combination determined by RT-qPCR. **B** The protein expression of PER2, Runx2 and OCN in AS-BMSCs treated with miR-22-3p mimic, oe-PER2, or in combination determined by Western blot analysis. **C** The viability of AS-BMSCs treated with miR-22-3p mimic, oe-PER2, or in combination detected by CCK-8 assay. **D** Mineralization of AS-BMSCs treated with miR-22-3p mimic, oe-PER2, or in combination detected using Alizarin red staining. **E** ALP content of AS-BMSCs treated with miR-22-3p mimic, oe-PER2, or in combination detected by ALP staining. *p < 0.05 vs. AS-BMSCs treated with NC mimic + oe-NC, #*p* < 0.05 vs. AS-BMSCs treated with miR-22-3p mimic + oe-NC. The cell experiment was repeated three times independently.

In order to further verify whether miR-22-3p influences the osteogenic differentiation of AS-BMSCs through PER2, miR-22-3p and PER2 were overexpressed in the AS-BMSCs. We found that miR-22-3p expression showed no alterations and that of PER2 was increased in the AS-BMSCs overexpressing PER2, while gain-of-function of miR-22-3p led to increased expression of miR-22-3p yet decreased expression of PER2. In addition, simultaneous overexpression of miR-22-3p and PER2 elevated PER2 expression without changing that of miR-22-3p than miR-22-3p-mimic treatment alone (Fig. 4A, B). Besides, a decline in the expression of Runx2 and OCN following PER2 overexpression, whereas an elevation was noted upon miR-22-3p overexpression. Combined treatment with miR-22-3p mimic and oe-PER2 caused lower expression of Runx2 and OCN than miR-22-3p mimic alone (Fig. 4B). Furthermore, cell viability was inhibited in AS-BMSCs overexpressing PER2. The promoting effect of miR-22-3p on the cell viability was abrogated by overexpressing PER2 (Fig. 4C). The mineralization nodules and ALP content were decreased in oe-PER2-treated AS-BMSCs, while the increase in mineralization nodules and ALP content caused by miR-22-3p mimic could be rescued by the overexpression of PER2 (Fig. 4D, E). These findings provided evidence verifying that miR-22-3p could enhance the osteogenic differentiation of AS-BMSCs by inhibiting PER2.

### PER2 blocked the Wnt/β-catenin signaling pathway by limiting Wnt7b

**Fig. 5:**
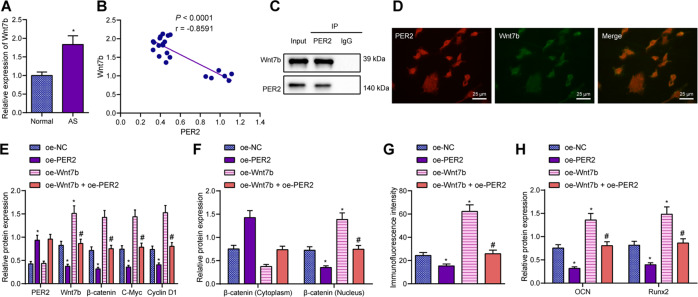
Effect of PER2 on the Wnt/β-catenin signaling pathway. **A** Wnt7b expression in the normal spinal ligaments of patients with spinal fractures (*n* = 6) and spinal ligaments of patients with AS (*n* = 16) determined by RT-qPCR. **B** The correlation between Wnt7b and PER2 expression in the spinal ligaments of patients with AS (*n* = 16) analyzed by Pearson’s correlation coefficient. **C** The interaction between Wnt7b and PER2 in AS-BMSCs detected by Co-IP. **D** The colocalization of Wnt7b and PER2 in AS-BMSCs detected by immunofluorescence staining, scale bar = 25 μm. **E** The protein expression of PER2, Wnt7b, and Wnt/β-catenin signaling pathway-related proteins (β-catenin, C-Myc, and Cyclin D1) in AS-BMSCs treated with oe-PER2 and/or Wnt7b measured by Western blot analysis. **F** The protein expression of β-catenin in the cytoplasm and nucleus of AS-BMSCs treated with oe-PER2 and/or Wnt7b detected by Western blot analysis. **G** Translocation of β-catenin to the nucleus of AS-BMSCs treated with oe-PER2 and/or Wnt7b detected by immunofluorescence staining. **H** The protein expression of Runx2 and OCN in AS-BMSCs treated with oe-PER2 and/or Wnt7b measured by Western blot analysis. *p < 0.05 vs. normal spinal ligaments/AS-BMSCs treated with oe-NC. #*p* < 0.05 vs. AS-BMSCs treated with oe-PER2. The cell experiment was repeated three times independently.

We initially hypothesized that PER2 could block the Wnt/β-catenin signaling pathway by means of suppressing Wnt7b in AS-BMSCs, ultimately inhibiting its osteogenic differentiation. We observed an enhancement in Wnt7b expression in the spinal ligaments of patients with AS relative to that in the normal spinal ligaments of patients with spinal fractures (Fig. 5A). Pearson’s correlation coefficient revealed the existence of a negative correlation between Wnt7b and PER2 in the spinal ligaments of patients with AS (Fig. 5B). Meanwhile, co-immunoprecipitation (Co-IP) results displayed that PER2 could bind to Wnt7b in AS-BMSCs (Fig. 5C). Colocalization of PER2 and Wnt7b was detected by cell immunofluorescence (Fig. 5D). Besides, the expression of Wnt7b, β-catenin, C-Myc and Cyclin D1 was reduced in AS-BMSCs treated with oe-PER2, while this effect could be reversed by overexpression of Wnt7b (Fig. 5E). Overexpression of Wnt7b could promote the translocation of β-catenin to the nucleus and reverse the inhibitory effect of oe-PER2 on the translocation of β-catenin to the nucleus (Fig. 5F, G). Moreover, expression of Runx2 and OCN was diminished in AS-BMSCs overexpressing PER2, and the results were opposite in AS-BMSCs treated with oe-Wnt7b (Fig. 5H).

### M2-EV-encapusulated miR-22-3p induced the osteogenic differentiation of AS-BMSCs by modulating PER2-mediated Wnt/β-catenin signaling pathway

**Fig. 6:**
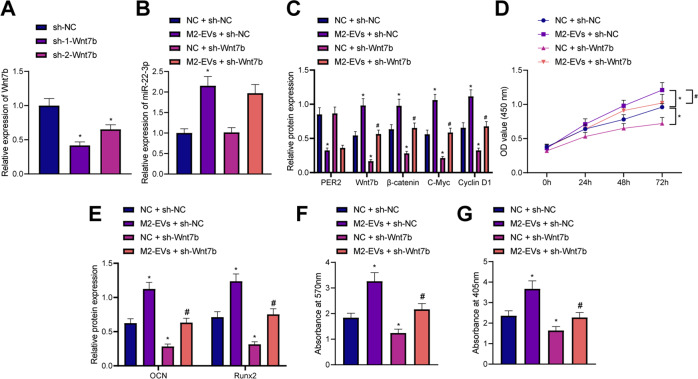
miR-22-3p shuttled by M2-EVs promoted the osteogenic differentiation of AS-BMSCs via the PER2/Wnt/β-catenin signaling. **A** Wnt7b mRNA expression in AS-BMSCs transfected with sh-1-Wnt7b or sh-2-Wnt7b determined by RT-qPCR. **B** miR-22-3p expression in AS-BMSCs treated with M2-EVs (100 µg/mL) and/or sh-Wnt7b measured by RT-qPCR. **C** The protein expression of PER2, Wnt7b, and Wnt/β-catenin signaling pathway-related proteins (β-catenin, C-Myc, and Cyclin D1) in AS-BMSCs treated with M2-EVs (100 µg/mL) and/or sh-Wnt7b measured by Western blot analysis. **D** The viability of AS-BMSCs treated with M2-EVs (100 µg/mL) and/or sh-Wnt7b detected by CCK-8 assay. **E** Expression of Runx2 and OCN in AS-BMSCs treated with M2-EVs (100 µg/mL) and/or sh-Wnt7b measured by Western blot analysis. **F** Mineralization of AS-MSCs treated with M2-EVs (100 µg/mL) and/or sh-Wnt7b detected using Alizarin red staining. **G** ALP content of AS-BMSCs treated with M2-EVs (100 µg/mL) and/or sh-Wnt7b detected by ALP staining. *p < 0.05, vs. AS-BMSCs treated with sh-NC/NC + sh-NC, #*p* < 0.05 vs. AS-BMSCs treated with M2-EVs + sh-NC. The cell experiment was repeated three times independently.

Next, to elucidate whether M2-EV-encapusulated miR-22-3p induced the osteogenic differentiation of AS-BMSCs through PER2-mediated Wnt/β-catenin signaling pathway, AS-BMSCs were transduced with lentivirus carrying sh-Wnt7b. After 5 days of screening stable cell lines, RT-qPCR was used to determine the transduction efficiency. The results exhibited that both sh-1-Wnt7b and sh-2-Wnt7b significantly reduced the expression of Wnt7b in AS-BMSCs, with sh-1-Wnt7b showing the better efficiency (Fig. 6A) and thus used for subsequent experiments. AS-BMSCs were treated with M2-EVs and/or sh-Wnt7b. Analysis concluded that the expression of miR-22-3p, β-catenin, C-Myc, and Cyclin D1 was increased, while that of PER2 was reduced in AS-BMSCs treated with M2-EVs. However, silencing of Wnt7b caused no changes in miR-22-3p and PER2 expression, but decreased that of β-catenin, C-Myc, and Cyclin D1. Meanwhile, silencing of Wnt7b could abolish the effect of M2-EVs on the expression of these factors (Fig. 6B, C). In addition, the viability of AS-BMSCs was enhanced upon coculture with M2-EVs, and the expression of PER2, Runx2, while OCN was decreased in the AS-BMSCs cocultured with M2-EVs, while the sh-Wnt7b induced opposite results and could reverse the effects of M2-EVs (Fig. 6D, E). Meanwhile, coculture with M2-EVs increased the mineralization and ALP content in AS-BMSCs, while treatment with shRNA targeting Wnt7b resulted in inhibition of the mineralization and ALP content, as well as rescuing the effect of M2-EVs (Fig. 6F, G).

### M2-EV-encapusulated miR-22-3p induced pathological osteogenesis in AS mice by regulating the PER2/Wnt/β-catenin signaling axis

**Fig. 7:**
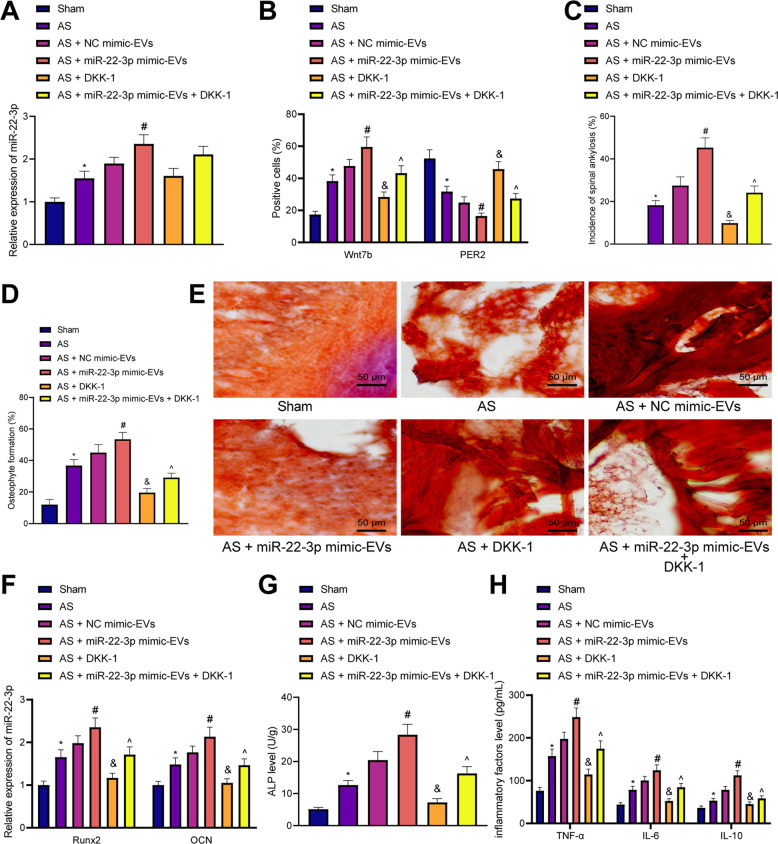
miR-22-3p shuttled by M2-EVs induced pathological osteogenesis in AS mice via the PER2/Wnt/β-catenin signaling. AS mice were treated with miR-22-3p mimic-EVs, DKK-1, or both. **A** miR-22-3p expression in the spinal tissues of AS mice determined using RT-qPCR. **B** PER2- and Wnt7b-positive cells in the spinal tissues of AS mice determined by immunohistochemical staining. **C** Micro-CT analysis of bone bridges (spine ankylosis) and ectopic new bone-formation rate in AS mice. **D** Vertebral osteophyte formation in AS mice observed using H&E staining. **E** Bone-bridge fusion between the vertebrae of AS mice detected using Alizarin red staining (scale bar = 50 μm). **F** Expression of Runx2 and OCN in the spinal tissues of AS mice measured using RT-qPCR. **G** ALP content in the serum of AS mice detected by sodium diphenyl phosphate microplate assay. **H** Levels of inflammatory factors IL-6, IL-10, and TNF-α in the serum of AS mice measured by ELISA. *p < 0.05 *vs*. sham-operated mice, #*p* < 0.05 vs. AS mice injected with NC mimic-EVs, and *p* < 0.05 vs. AS mice, ^*p* < 0.05 vs. AS mice injected with miR-22-3p mimic-EVs. *n* = 6.

In order to evaluate whether miR-22-3p in M2-EVs could induce the pathological osteogenesis in vivo, AS mouse models were established using proteoglycan and Freund’s complete adjuvant, and treated with 100 μg of NC mimic-EVs, miR-22-3p mimic-EVs, or Wnt protein inhibitor Dickkopf-1 (DKK-1), via the tail vein. It was evident that miR-22-3p expression was increased in the spinal tissues of AS mice. The expression of miR-22-3p showed no changes in the spinal tissues of DKK1-treated AS mice. In comparison with AS mice treated with NC mimic-EVs, miR-22-3p expression also elevated in the spinal tissues of AS mice injected with miR-22-3p mimic-EVs. Combined treatment with miR-22-3p mimic-EVs and DDK1 did not alter the expression of miR-22-3p in the spinal tissues of AS mice relative to treatment with miR-22-3p mimic-EVs (Fig. 7A). The number of PER2-positive cells was decreased, while the number of Wnt7b-positive cells was increased in the spinal tissues of AS mice, while DKK1 treatment led to decreased Wnt7b-positive cells. In the spinal tissues of AS mice injected with miR-22-3p mimic-EVs, the number of PER2-positive cells was decreased, while that of Wnt7b positive cells was increased. Conversely, the number of positive cells of Wnt7b was diminished in the spinal tissues of AS mice treated with miR-22-3p mimic-EVs + DKK-1 when compared with the AS mice treated with miR-22-3p mimic-EVs alone (Fig. 7B).

Furthermore, the results of microcomputed tomography (Micro-CT) (Fig. 7C), hematoxylin and eosin (H&E) staining (Fig. 7D), and alizarin red staining (Fig. 7E) revealed that the AS mice had significant bone bridges (spine ankylosis), ectopic new bone formation between the vertebrae, distinct osteophytes, and bone-bridge fusion. Treatment with DKK-1 was found to reduce spinal rigidity, new ectopic bone formation, and intervertebral osteophyte formation, and delayed bone-bridge fusion. The spinal rigidity, ectopic new bone formation, intervertebral osteophyte formation, and bone-bridge fusion were increased in AS mice injected with miR-22-3p mimic-EVs, the effect of which was reversed by DKK-1. We also observed an enhancement in the expression of Runx2 and OCN in the spinal tissues, as well as in the ALP content and levels of IL-6, IL-10, and TNF-α in the serum of AS mice compared with the sham-operated mice. Conversely, treatment with DKK-1 led to opposite results. In the presence of miR-22-3p mimic-EVs, the expression of Runx2 and OCN, along with ALP content and levels of IL-6, IL-10, and TNF-α in the serum of AS mice, was increased, while DKK-1 reversed the effects of miR-22-3p mimic-EVs (Fig. 7F–H).

## Discussion

AS represents a commonly occurring autoimmune disease that arises in the setting of pathological osteogenesis [4]. Existing literature has highlighted the abnormal function of osteoclasts as a chief factor in the pathogenesis of osteogenesis of AS [17]. In recent years, the disability rate of patients with AS has exhibited increases owing largely to a lack of specific therapeutic targets for osteogenesis [18]. A recent study concluded that MSCs contribute to bone formation while linking it to the pathological osteogenesis observed in AS [19]. The more specific mechanisms that underpin the abnormal osteogenic differentiation observed in AS are not fully understood, with the objective of our study centered around an investigation into the effect of EV-encapsulated miR-22-3p from M2 macrophage, PER2, and Wnt/β-catenin on the osteogenic differentiation of MSCs in AS and their accompanied mechanisms. We unveiled that miR-22-3p shuttled by M2-EVs could be delivered into BMSCs, whereby miR-22-3p exerted stimulatory effects on the osteogenic differentiation of AS-BMSCs in AS via activation of the Wnt/β-catenin signaling pathway by regulating PER2 both in vitro and in vivo.

Our initial observations revealed that EVs derived from M2 macrophages promoted osteogenic differentiation of BMSCs in AS. Previous literature has provided evidence implicating the dysfunctional osteogenic differentiation of MSCs with disorders of bone metabolism in rheumatic diseases [20]. EVs have been well documented as crucial regulators of intercellular communication and disease development by transporting a complex cargo of biologically active factors to target cells [21]. EVs possess the capacity to influence a wide array of processes in the body, including that of inflammation [22]. EVs continue to attract attention as regulators of bone remodeling, such as rheumatoid arthritis, osteoarthritis, and osteoporosis [23, 24]. A previous study has suggested that M2 macrophage-derived exosomal miR-5106 is capable of inducing BMSCs to influence the process of osteogenesis [25]. The current study provided evidence verifying the notion that miR-22-3p from M2 macrophages transferred into BMSCs via EVs to induce the osteogenic differentiation of AS-BMSCs. EVs have been previously reported to transport various small biological molecules, including miRNAs, to surrounding cells [26]. Evidence continues to be reported highlighting the elevated expression of miR-22-3p in AS [13]. Notably, the expression of miR-21 has been reported to be increased in EVs and cell lysate isolated from M2-polarized macrophages [27]. These findings are the same with ours, whereby miR-22-3p shuttled by M2-EVs enhanced the osteogenic differentiation of BMSCs in AS.

Moreover, our experimental results unfolded that miR-22-3p could target PER2 and inhibit the expression of PER2. PER2 is a tumor suppressor and is upregulated in breast cancer [28]. PER2 possesses inhibitory action in the osteogenic differentiation of BMSCs [29]. Our data further confirmed that PER2 could repress Wnt7b to block the Wnt/β-catenin signaling pathway. Similarly, PER2 can block the Wnt/β-catenin signaling pathway to limit the stemness of glioma stem cells [16]. Both in vitro and in vivo experiments demonstrated that miR-22-3p shuttled by M2-EVs could reduce the expression of Runx2, OCN, and ALP, and mineralization to trigger the osteogenic differentiation of AS-BMSCs by suppressing PER2 via activation of the Wnt/β-catenin signaling pathway. Wnt/β-catenin signaling pathway has been reported to bear the critical responsibility of AS pathogenesis [30]. Besides, activation of the Wnt signaling pathway contributes to osteogenic differentiation and bone formation in AS mice [31]. However, relatively few studies have explored the underlying mechanism of miR-22-3p in the activation of the Wnt/β-catenin signaling pathway by inhibiting PER2 to induce the osteogenic differentiation of BMSCs in AS.

**Fig. 8:**
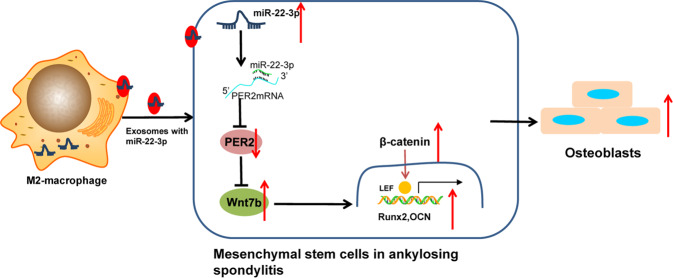
Effects of M2 macrophage-derived extracellular vesicles (EVs) on the development of ankylosing spondylitis (AS) through by transferring of microRNA-22-3p. The mechanism diagram illustrating that miR-22-3p shuttled by M2-EVs promoted the osteogenic differentiation of AS-BMSCs and the resultant pathological osteogenesis in AS mice via activation of the Wnt/β-catenin signaling pathway by targeting PER2.

In short, our experimental evidence pointed that the transfer of miR-22-3p via M2-EVs altered the PER2 expression to regulate the Wnt/β-catenin signaling pathway, which ultimately led to the osteogenic differentiation of AS-BMSCs (Fig. 8). Our findings provide a promising basis for novel effective therapeutic strategies capable of treating bone metastasis in AS. Due to the limited supporting literature, the roles of miR-22-3p shuttled by M2-EVs, PER2, and Wnt/β-catenin, as well as their interactions in the osteogenic differentiation of BMSCs in AS, should be discussed in more detail, which will need to be monitored in a rigorous manner and reported appropriately in the future clinical trials.

## Materials and methods

### Study subjects

Spinal ligaments were collected from 16 patients with AS who had been previously diagnosed and received surgical intervention (11 males and 5 females with a mean age of 34.5 years) at the Department of Spine and Osteopathy Ward in The First Affiliated Hospital of Guangxi Medical University between May 2018 and January 2019. All of these patients were confirmed to be exhibiting inflammatory lower back pain, significant ossification lines in the sacroiliac joint observed by X-ray pelvic radiographs, elevated levels of C-reactive protein, and erythrocyte-sedimentation rate, and were confirmed to be HLA-B27 positive. All patients fulfilled the 1984 revised New York Criteria of American Rheumatism Association for AS [32]. At the same time, normal spinal ligaments were collected from 6 patients (4 males and 2 females with a mean age of 32.5 years) with spinal fractures who had been admitted to the Department of Spine and Osteopathy Ward at The First Affiliated Hospital of Guangxi Medical University between March 2018 and February 2019 and served as the control. RNA samples and protein extraction were followed by prompt preservation in liquid nitrogen, with the samples for immunohistochemistry fixed with 10% neutral formalin solution and then paraffin-embedded.

### Isolation, culture, and identification of human BMSCs

Next, to diminish the influence of other treatments, all treatments were stopped 14 days prior to BM puncture. After BM puncture, human BMSCs were immediately isolated and purified from BM aspiration of patients with AS by density-gradient centrifugation. Next, BMSCs were incubated in Dulbecco’s modified Eagle’s medium (DMEM) (10099141, Gibco, Carlsbad, CA, USA) containing 10% fetal bovine serum (FBS) (10099141, Gibco) with 5% CO_2_ at 37 °C, and the medium was renewed every 3 days. Upon reaching 70−80% confluence, the BMSCs at passage 3−5 were employed for subsequent experiments.

BMSCs were identified by flow cytometry based on cellular immune phenotypes. After washing with phosphate-buffered saline (PBS), BMSCs were cultured with fluorescein isothiocyanate (FITC)-conjugated CD44 (#43675, 1:20, CST, USA), FITC-conjugated CD45 (#14579, 1:50, CST), phycoerythrin (PE)-conjugated CD14 (#44947, 1:20, CST), PE-conjugated human leukocyte antigen DR (HLA-DR) (#78397, 1:20, CST), FITC-conjugated CD90 (ab226, 1:500, Abcam, Cambridge, UK), FITC-conjugated CD105 (ab184667, 1:100, Abcam), and PE-conjugated CD73 (ab157335, 1:100, Abcam) followed by additional PBS washing. After suspension, cell sorting was conducted using BD Biosciences flow cytometer. Flow cytometry validated the successful isolation of BMSCs (Figure S3).

### Osteogenic differentiation of AS-BMSCs

AS-BMSCs were seeded into 12-well plates (1.5 × 10^4^ cells/cm^2^), followed by incubation in a DMEM growth medium (GM) containing 10% FBS. Upon reaching 80% cell confluence, the culture medium was substituted with the osteogenic differentiation medium of MSCs (OM, GuxMX-90021, Cyagen, USA) (including 10% FBS, 1% penicillin-streptomycin, 1% glutamine, 0.2% ascorbate, 1% glycerophosphate, 0.01% dexamethasone, 100 IU/mL penicillin, and 100 IU/mL streptomycin). The cells were treated accordingly and fixed with 4% paraformaldehyde (PFA) after 14 days, after which osteogenesis was evaluated using ALP staining and alizarin red staining. AS-BMSCs (day 0 of induction) cultured in GM were regarded as the control.

### Culture, ioslation and identification of M2 macrophages

Human mononuclear macrophage cell lines (THP-1, American Type Culture Collection (ATCC, VA, USA)) cultured in Roswell Park Memorial Institute-1640 medium containing 10% FBS were cultured in an incubator at constant controlled temperature of 37 °C under full humidity with 5% CO_2_. Upon reaching 90% confluence, all cell lines used in 1:3–1:4 digestive passage were verified using short tandem-repeat analysis in addition to being confirmed to be free of mycoplasma contamination. THP-1 cells were treated with 100 ng/mL polarized 12-myristate 13-acetate (PMA, P8139, Sigma-Aldrich Chemical Company, St Louis, MO, USA) for 48 h and then incubated for 24 h without PMA to differentiate into macrophages. After that, the cell morphology changed significantly. The ratio of cytoplasm was significantly increased under a microscope, and the cell adherence was better. Next, the undifferentiated THP-1 cells were removed from the culture dish using the adherence characteristics. Purified macrophages were treated with 20 ng/mL interleukin-4 (IL-4) (AF-200-04-5, Peprotech, Rocky Hill, NJ, USA) for 72 h to facilitate differentiation into M2 phenotype.

The surface markers of M2 macrophages were identified using flow cytometry. First, specific antibodies to F4/80 (#123107, 1:2000, Biolegend, San Diego, CA, USA) and CD206 (#141705, 1:1000, Biolegend) were used to isolate M2 macrophages from macrophages. Next, specific antibodies to F4/80 (#123107, 1:2000, Biolegend) and CD206 (#141705, 1:1000, Biolegend) were applied for the identification of the isolated M2 macrophages. Data analysis was implemented by means of FlowJo V10.6.1 software.

### Extraction and identification of M2-EVs

FBS was centrifuged (100,000 × *g*) for 18 h to remove EVs in the serum. After reaching 80–90% cell confluence, the sample was rinsed twice using PBS followed by removal of the culture supernatant. The medium was renewed with 10% EV-depleted FBS for another 48-h incubation under CO_2_ at 37 °C. The cell supernatant was collected for isolation of EVs by means of ExoQuick exosome extraction kit (ExoQ5A-1, SBI, USA). The precipitation formed following centrifugation was washed using PBS, resuspended, and stored at −80 °C. The morphology of EVs was identified using a transmission electron microscope (TEM; EFI, TECNAI G2), and the size of EVs was analyzed using nanometer instruments (Malvern Instruments, Malvern, UK) by dynamic light scattering (DLS). The expression of EV-surface markers CD63 (ab1318, 1:500, Abcam), CD81 (ab79559, 1:1000, Abcam), TSG-101 (ab125011, 1:1000, Abcam), and calnexin (ab133615, 1:1000, Abcam) was evaluated using Western blot analysis.

### EV-uptake assay

The purified M2-EVs were labeled utilizing the PKH26 red fluorescence kit (PKH26GL-1kt, Sigma-Aldrich). M2-EVs and PKH26 were suspended in 1 mL of diluent C for labeling purposes. Following incubation for 1 min, the labeling process was terminated using 1% bovine serum albumin (BSA) (Bovogen, Melbourne, Australia) of an identical volume, followed by three PBS washes containing Amicon ultrafilter (10-kda cutoff, Millipore, MA, USA) and resuspension with PBS. Next, PKH26-labeled M2-EVs were cocultured with AS-BMSCs for 12 h. The cells were fixed using 4% PFA and the nuclei were stained with 4′,6-diamidino-2-phenylindole (DAPI) (blue), after which the cytoskeleton was stained green using F-actin (ab130935, Abcam). Then, the cells were incubated at 37 °C for 12 h. The uptake of M2-EVs by cells was visualized under a confocal microscope (Zeiss Meta 510, Thornwood, NY, USA).

M2 macrophages were transduced with Cy3–miR-22-3p lentivirus (GenePharma Co. Ltd., Shanghai, China) in the serum-free medium utilizing the lipo3000 kit (L3000001, Invitrogen, Carlsbad, CA, USA) for 6 h, and further cultured in 10% EV-free serum medium for 48 h. The cell supernatant was collected for isolation of EVs using the aforementioned method, which was supplemented into AS-BMSCs. Then, the samples were subsequently fixed with 4% PFA, washed with PBS, and stained, and the uptake of EVs (red light) carrying Cy3–miR-22-3p by AS-BMSCs was observed under the confocal microscope (Zeiss Meta 510, Thornwood, NY, USA) with AS-BMSCs transduced with lentivirus carrying miR-NC as NC.

### Cell transfection and grouping

Prior to transfection, the cells were seeded in 6-well plates (1 × 10^5^ cells/well), and cultured routinely. Upon 75% confluence, the cells were transfected with miR-22-3p mimic, miR-22-3p inhibitor, and gene-overexpression vectors in light of the Lipofectamine 2000 transfection reagent (11668-019, Invitrogen). Briefly, 4 μg of the target plasmid or 200 pmol miR-22-3p mimic, miR-22-3p inhibitor, and 10 μL Lipofectamine 2000 were diluted with 250 μL of serum-free Opti-MEM (Gibco). After mixture, the sample was cultured in a 5% CO_2_ incubator at 37 °C. After 6 h, the medium was refreshed with complete medium, and the cells were further cultured for 48 h and collected. The cells were transduced with lentivirus carrying shRNA at a titer of 1 × 10^9^ TU/mL. After 48 h, the medium was refreshed with complete medium containing 2 μg/mL puromycin (A1113803, Gibco) or 500 μg/mL G418 (11811023, Gibco), and the cells continued to culture for 3 days to screen the stable cell lines. Lentivirus was purchased from Invitrogen. All plasmids and miR-22-3p mimic and miR-22-3p inhibitor were synthesized by Shanghai Genechem Co., Ltd. (Shanghai, China).

Group 1: NC (AS-BMSCs were added with PBS and transfected with NC mimic), miR-22-3p mimic (AS-BMSCs were added with PBS and transfected with miR-22-3p mimic), M2-EVs + iNC (AS-BMSC medium was added with M2-EVs and transfected with NC inhibitor), and M2-EVs + miR-22-3p inhibitor (AS-BMSC medium was added with M2-EVs and transfected with miR-22-3p inhibitor).

Group 2: NC mimic + oe-NC (AS-BMSCs were transfected with NC mimic and oe-NC), NC mimic + oe-PER2 (AS-BMSCs were transfected with NC mimic and oe-PER2), miR-22-3p mimic + oe-NC (AS-BMSCs were transfected with miR-22-3p mimic and oe-NC), and miR-22-3p mimic + oe-PER2 (AS-BMSCs were transfected with miR-22-3p mimic and oe-PER2).

Group 3: oe-NC (AS-BMSCs were transfected with oe-NC), oe-PER2 (AS-BMSCs were transfected with oe-PER2), oe-Wnt7b (AS-BMSCs were transfected with oe-Wnt7b), and oe-Wnt7b + oe-PER2 (AS-BMSCs were transfected with oe-Wnt7b and oe-PER2).

Group 4: NC + sh-NC (sh-NC-treated AS-BMSCs were incubated with PBS for 3 days), M2-EVs + sh-NC (sh-NC-treated AS-BMSCs were incubated with M2-EVs for 3 days), NC + sh-Wnt7b (sh-Wnt7b-treated AS-BMSCs were incubated with PBS for 3 days), and M2-EVs + sh-Wnt7b (sh-Wnt7b-treated AS-BMSCs were incubated with M2-EVs for 3 days).

### CCK-8 assay

Following varying treatments, 10 μL CCK-8 at 0 h, 24 h, 48 h, and 72 h was added to the AS-BMSCs for 1-h culturing in accordance with the CCK-8 kit instructions (C0037, Beyotime). The absorbance value was tested utilizing an enzyme standard instrument (Bio-Rad Laboratories, Hercules, CA, USA) at 450 nm. A growth curve was constructed based on the absorbance values in order to determine cell viability.

### Coculture of M2 macrophages and AS-BMSCs

M2 macrophages and AS-BMSCs were detached using trypsin, centrifuged (1000 × *g*) for 5 min, and subsequently resuspended using 3 mL of medium with 10 μL suspension counted under a cell-counting plate. M2 macrophages and AS-BMSCs were respectively placed into two Transwell chambers of a 6-well plate for cocultivation for 4–5 d with the upper chamber supplemented with 10% serum DMEM, and the lower chamber with 15% serum DMEM. The fluid was refreshed every 1–2 days. All medium in the upper and lower chambers was renewed at the same time. After 72 h, when the cell density reached over 80%, AS-BMSCs were collected and washed with PBS. Upon reaching 80% confluency, M2 macrophages were incubated with EV inhibitor GW4869 (final concentration of 5 μm) for 48 h.

### RT-qPCR

Total RNA extraction was implemented utilizing TRIzol reagent (15596-018, Solarbio Science & Technology Co., Ltd., Beijing, China), with the purity and RNA concentration subsequently determined. RNA was reversely transcribed into cDNA with the help of the PrimeScript RT reagent Kit (RR047A, Takara, Japan). RNA was extracted from the M2-EVs using the Exosomal RNA Isolation Kit (NGC-58000, Norgen Biotek), allowing for the cDNA of the miRNAs to be synthesized utilizing the TaqMan microRNA assay Kit (Applied Biosystems, Foster City, USA). SYBR Green Master Mix (Life Technologies, Carlsbad, CA, USA) and ABI PRISM 7500 RT-PCR system (Applied Biosystems) were employed for RT-qPCR detection and quantitative analysis of gene expression. All primers were designed and synthesized by Shanghai Balog Biotechnology Co., Ltd. (Shanghai, China). The primer sequences used are illustrated in Table S1. The final concentration of primers used was 2 μM. U6 was regarded as the internal reference for miR-22-3p, and GAPDH for the remaining genes. Analysis of gene relative expression was performed using the 2^–ΔΔCt^ method.

In Figure S3, AS-BMSCs were incubated with different concentrations of M2-EVs and the relative expression of miR-22-3p in the AS-BMSCs was detected. M2-EVs were first treated with 0.4 μg/mL RNase A, 0.1 mg/ml Proteinase K, and 5% Triton X-100 at 37 °C for 20 min, and then co-incubated with AS-BMSCs at different concentrations for 48 h, followed by RT-qPCR detection.

### Western blot analysis

Total protein extraction was implemented using radio-immunoprecipitation assay (R0010, Solarbio) containing phenylmethylsulfonyl fluoride. The protein concentration was detected by bicinchoninic acid kit (C503021-0500, Shanghai Sangon Biological Engineering Technology & Services Co., Ltd., Shanghai, China). Next, 50 μg of protein was separated with sodium dodecyl sulfate–polyacrylamide gel electrophoresis (SDS-PAGE), and electrotransferred onto a polyvinylidene fluoride membrane (Merck Millipore, USA). Membrane blockade was conducted using 5% BSA on a shaking table for 1 h, and then the membrane was incubated overnight at 4 °C with the following primary antibodies: rabbit anti-PER2 (ab179813, 1:5000), Runx2 (ab23981, 1:1000), OCN (ab93876, 1:500), β-catenin (ab32572, 1:5000), C-Myc (ab32072, 1:1000), Cyclin D1 (ab16663, 1:4000), Wnt7b (ab94915, 1:1000), GAPDH (ab8245, 1:5000, internal reference), and histone H3 (ab1791, 1:1000). All antibodies were provided by Abcam. Subsequently, the membrane was incubated with the horseradish peroxidase-labeled goat anti-rabbit secondary antibody immunoglobulin G (IgG) (ab6721, 1:5000, Abcam) for 1 h. Following three TBST washes (15 min per wash), the membrane was added with the luminescent solution. The results were analyzed by Bio-Rad gel imaging analysis system (Bio-Rad, Hercules, CA, USA) and Image J.

### Alizarin red staining

AS-BMSCs were cultured in osteogenic medium (OM) for 14 days, washed twice with PBS, fixed with 10% formalin for 15 min, and stained with the use of 1 mL of 0.5% Alizarin red staining solution for 15 min. After rinsing with distilled water for 5 min, the red calcified nodules were visualized using a charge-coupled microscope. For quantification of mineralization, staining was dissolved in 100 mmol/L cetylpyridinium chloride for 30 min and tested at a 570 nm of absorbance value.

### ALP staining

Cells cultured for 7 days in OM were utilized for ALP staining and quantitative analysis by means of the NBT/BCIP staining kit (C3206, Beyotime). Briefly, the cells were washed twice with PBS, fixed with 10% formalin for 15 min, and treated with BCIP/NBT substrate for 24 h. Charge-coupled microscope was used to analyze the colorimetric changes and the stained cells were imaged using a scanner. The absorbance was subsequently measured at 405 nm

### Bioinformatics analysis

The TargetScan database [33] was explored for prediction of miRNA target gene. AS-related expression dataset GSE11886 (8 AS samples and 9 normal samples) was searched through the Gene Expression Omnibus database, followed by differential analysis, with the “limma” software package in R [34] for finding the differentially expressed genes with |logFoldChange∣ > 1 and false-discovery rate < 0.05 as the threshold.

### Dual-luciferase reporter gene assay

HEK293T cells (ATCC) were cultured in DMEM containing 10% FBS with 5% CO_2_ and saturated humidity at 37 °C. The PER2 sequence containing miR-22-3p-binding site and mutation-binding site was cloned into pGL3-Promoter luciferase-reporter vector (Promega, Madison, WI, USA) in order to construct PER2-WT and PER2-MUT reporter vectors. Next, miR-22-3p mimic or NC mimic was cotransfected into HEK-293T cells with the aforementioned reporter vectors using Lipofectamine 3000 reagent (Invitrogen) for 48 h. Luciferase activities were measured based on the instructions of the dual-luciferase reporter kit (Promega).

### RNA pull-down assay

miR-22-3p mimic-transfected AS-BMSCs were further transfected with 50 nM biotinylated PER2-WT and 50 nM biotinylated PER2-MUT for implementing RNA pull-down assay [35]. miR-22-3p enrichment was examined by RT-qPCR.

### RIP assay

The binding between miR-22-3p and PER2 was analyzed using RIP kit (#17-700, Millipore, Billerica, MA, USA) [36] with rabbit anti-human AGO2 (2 μg, ab32381, Abcam, mixed at room temperature for 30 min) and rabbit anti-human IgG (2 μg, ab109489, Abcam, used as a NC).

### Co-IP assay

The cells were split in a lysis buffer containing 100 mM Tris-HCl (Sangon) (pH 7.4), 150 mM NaCl, 10% glycerol, 0.5% Triton X-100 (Sangon), and protease inhibitors. The cell lysate was centrifuged for supernatant-collection purposes. Protein A/G Sepharose beads were added to the supernatant to remove nonspecific binding in advance. Then PER2 antibody (ab179813, 1:5000, Abcam) was added and incubated with the lysate at 4 °C. After overnight incubation, protein A/G sepharose beads were added for 1 h, washed 4 times using lysis buffer, eluted with SDS-PAGE sample buffer, and analyzed by Western blot with PER2 antibody and Wnt7b antibody.

### Immunofluorescence staining

AS-BMSCs were subjected to fixation utilizing 4% PFA, PBS rinsing, and permeabilization with 0.1% Triton X-100. After blocking with 5% BSA, the cells were incubated with primary antibodies against β-catenin (ab32572, 1:250, Abcam), PER2 (ab179813, 1:200, Abcam), and Wnt7b (A7746, 1:200, ABclonal) at 4 °C for 20 h. Then, the AS-BMSCs were incubated with Alexa Fluor®488-conjugated secondary antibody goat anti-rabbit IgG (H&L, ab150081, 1:500, Abcam) containing fluorescence at 37 °C for 1 h under conditions void of light, and counterstained with DAPI for 10 min. Finally, a confocal fluorescence microscope (Leica Microsystems GmbH) was used to observe the protein colocalization in cells in at least 5–10 different visual fields. ImageJ software was employed to analyze and calculate the intensity of the overlapping fluorescence signals of β-catenin and DAPI in the fluorescence pictures of each group of cells (no less than 50 cells). The mean fluorescence intensity was used to evaluate the translocation of β-catenin to the nucleus.

### AS mouse model establishment

Thirty-six male BALB/C mice (aged 5–7 weeks and weighing 18–22 g, Vital River Laboratory Animal Technology Co., Ltd., Beijing, China) were randomly divided into 6 groups (*n* = 6). Prior to experiments, the mice were raised in a specific pathogen free environment with comfortable temperature, sterile feed, and drinking water, and an alternating 12 h day–night cycle for 7 days.

Preparation of mouse model of AS [37, 38]: The mice were injected intraperitoneally with 100 mg of cartilage proteoglycan (Sigma-Aldrich) at 0, 3, and 6 weeks, where the first and third proteoglycan injections utilized complete Freund’s adjuvant (Difco, Detroit, Michigan), and the second proteoglycan injection used incomplete Freund’s adjuvant (Difco). The mice in the sham-operated group were administered with an identical volume of PBS solution. Micro-CT scans were implemented to assess the extent of spinal lesions in order to verify the successful establishment of AS models. After 21 days, the degree of joint swelling and joint pathology in mice was used as the evaluation criteria for the success of the model establishment [39].

Following successful model establishment, the mice were injected with M2 macrophages treated with NC mimic-EVs, miR-22-3p mimic-EVs, DKK-1 (10 µg/g, R&D, Minneapolis, MN, USA) [39], or an equal volume of PBS alone or in combination via tail vein for three consecutive days. After 30 days of treatment, the mice were euthanized under deep anesthesia, with the spinal specimens dissected and fixed in 4% PFA for CT and histological analysis.

### Immunohistochemistry

Paraffin-embedded sections were deparaffinized, and the operation was carried out according to conventional immunohistochemistry methods. The sections were incubated with the primary antibodies Wnt7b (ab94915, 1:200, Abcam) and PER2 (AF7728, 1:500, Beyotime) and the secondary antibody IgG (ab150083, 1:100, Abcam). Nonspecific normal IgG was used as the NC. Five lesion areas were randomly selected under a microscope (200× or 400×) with the number of positive staining cells determined. The positive-cell value and standard deviation were used for identification, followed by statistical analysis.

### Micro-CT analysis

The lumbar vertebrae (spine segments, including intervertebral disks and adjacent endplates) of mice in each group were taken and fixed with 4% PFA. The Skyscan 1176 micro-CT instrument (Bruker microCT, Kontich, Belgium) was applied for micro-CT analysis. The instrument parameter settings were AI 0.5-mm filter, source current 500 mA, source voltage 50 kV, rotation step length 0.4°, and pixel size 9 µm. After the scan, segmented data and use of Micro-CT Ray V3.0 software (Scanco Medical) were used to reconstruct a three-dimensional image.

### H&E staining

After the experiment, the mice were euthanized using a lethal dose of anesthesia, and the surrounding spinal tissue was dissected to ensure its integrity. The dissected samples were fixed overnight with 10% formalin, decalcified with ethylenediaminetetraacetic acid solution, dehydrated with ethanol, and embedded with paraffin, and the spinal tissues were sectioned (5 μm).

The tissue sections were dewaxed, hydrated, stained with hematoxylin for 5 min, subsequently differentiated with ethanol in alkaline water, stained with eosin for 15 s, followed by dehydration with 95% ethanol for 2 min (2 times), and dehydration with 100% ethanol for 3 min (2 times). The sections were subsequently cleared with xylene and sealed with neutral glue. Pathological changes in the cartilage were then observed under a light microscope and photographed (NIKON CORPORATION, Tokyo, Japan).

### Determination of ALP levels in the serum of mice

After the experiment, the mice were deeply anesthetized, followed by collection of the whole blood by cardiac puncture. Following incubation for 30 min, the serum was attained after centrifugation (5000 rpm) for 10 min. In light of the Sodium Diphenyl Phosphate Microplate Assay Kit (SNM138, Biolab, Beijing, China), the levels of ALP were determined.

### ELISA

The levels of inflammatory factors IL-6, IL-10, and tumor necrosis factor-α (TNF-α) were detected in strict accordance with the instructions of the IL-6 ELISA Kit (PI326, Beyotime), IL-10 ELISA Kit (PI522, Beyotime), and TNF-α ELISA Kit (PI512, Beyotime), respectively.

### Statistical analysis

Data analysis was implemented using SPSS 21.0 software (IBM, Armonk, NY, USA). Measurement data appeared as the mean ± standard deviation. Data with normal distribution and homogeneity of variance between two groups were evaluated utilizing unpaired *t*-test. Data comparisons among multiple groups were analyzed using one-way analysis of variance (ANOVA) or repeated-measures ANOVA, followed by Tukey’s post hoc test. Wilcoxon rank-sum test was selected for data with skewed distribution and defect variances. Pearson’s correlation coefficient was implemented for correlation analysis. *p* < 0.05 or *p* < 0.0001 was concluded as statistically significant.

## Supplementary information

Supplementary Information

## Data Availability

The datasets generated/analyzed during the current study are available.
