# Supplementary material for: Transfer of microRNA-22-3p by M2 macrophage-derived extracellular vesicles facilitates the development of ankylosing spondylitis through the PER2-mediated Wnt/β-catenin axis
Source: Cell Death Discov. 2022 May 23;8:269. doi: 10.1038/s41420-022-00900-1 (PMC9126881; doi:10.1038/s41420-022-00900-1)
Supplement: Supplementary file 1 — Supplementary Information [file 41420_2022_900_MOESM1_ESM.pdf]

Supplementary Information

Figure S1 Microscopic images of THP-1 cell differentiation into macrophages before and after induction. Scale bar = 50 μm. The cell experiment was repeated 3 times independently.

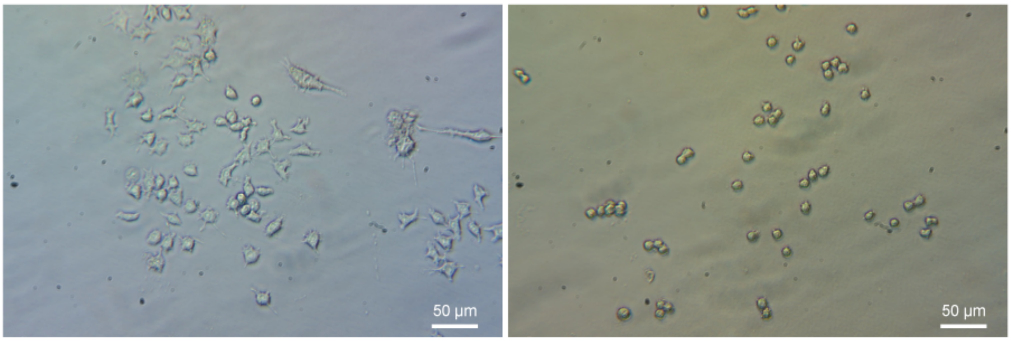

Figure S2 miR-22-3p expression in AS-BMSCs co-cultured with M2-EVs at different concentrations following treatment with RNase A, Proteinase K and Triton X-100 determined by RT-qPCR. * $p < 0.05$, *vs.* PBS. # $p < 0.05$, *vs.* 25 μg/mL M2-EVs. & $p < 0.05$, *vs.* 50 μg/mL M2-EVs. The cell experiment was repeated 3 times independently.

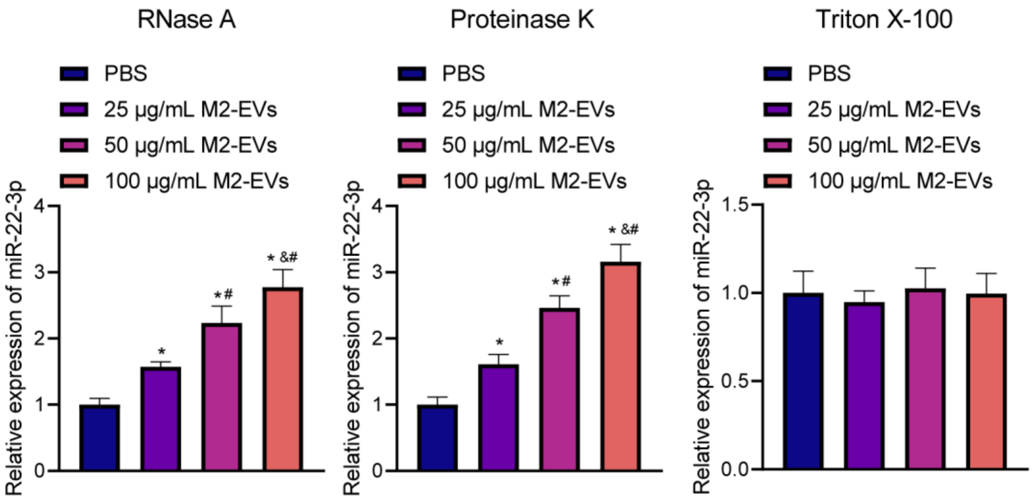

Figure S3 Flow cytometric analysis of BMSC surface antigen CD73, CD90, CD44 and CD105 (positive) and non-BMSC surface antigen HLA-DR, CD45 and CD14 (negative). The cell experiment was repeated 3 times independently.

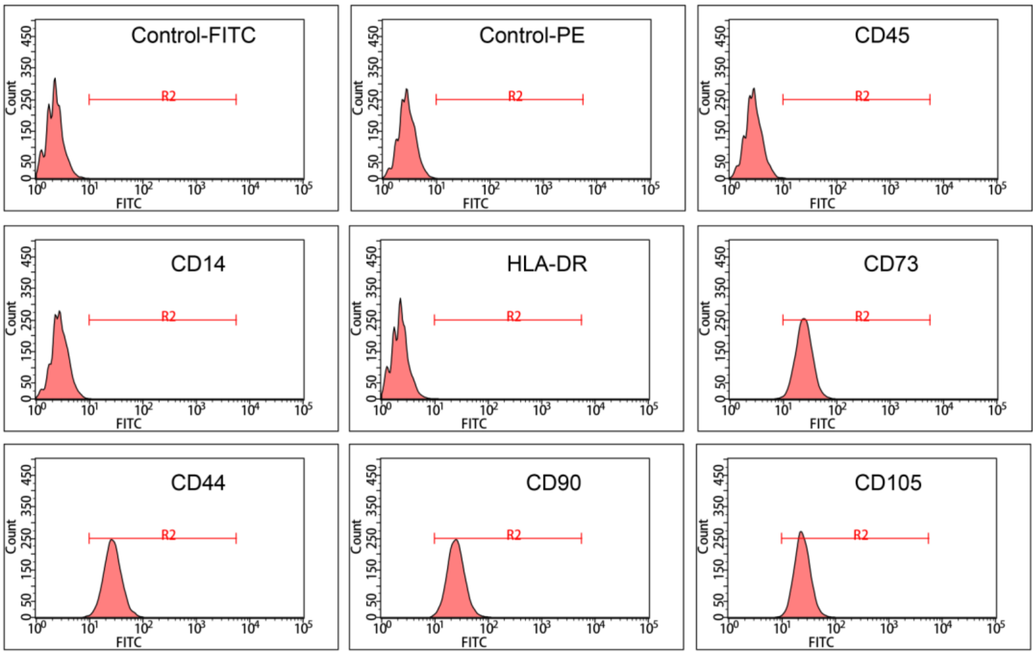

Table S1 RT-qPCR primer sequences

Genes	Primer sequences (5'-3')
miR-22-3p (human & mouse)	F: 5'-GGTTAAGATGCCAGTTGAA-3'<br>R: 5'-GGCAGAGGGCAACAG-3'
Runx2 (human)	F: 5'-CGCCACCACTCACTACCACAC-3'<br>R: 5'-TGGATTTAATAGCGTGCTGCC-3'
Runx2 (mouse)	F: 5'-CTCACAAACAACCACAGAACCACAA-3'<br>R: 5'-CTTGCAGCCTTAAATGACTCGG-3'
OCN (human)	F: 5'-TCACACTCCTCGCCCTATTG-3'<br>R: 5'-CTCTTCACTACCTCGCTGCC-3'
OCN (mouse)	F: 5'-TTGGCTTCCTGGGCTGGTAT-3'<br>R: 5'-GGAGAGCTCTTCACACCGTT-3'
U6 (human & mouse)	F: 5'-CTCGCTTCGGCAGCACA-3'<br>R: 5'-AACGCTTCACGAATTTGCGT-3'
PER2 (human)	F: 5'-GCTGGACTCCTCGGCTTGAA-3'<br>R: 5'-GGAACGAAGCTTTCGGACCT-3'
GAPDH (human)	F: 5'-CATGGCAAATTCCATGGCACCGTC-3'<br>R: 5'-CCCTTTTGGCTCCCCCCTGCA-3'
GAPDH (mouse)	F: 5'-GGGTCCCAGCTTAGGTTCATC-3'<br>R: 5'-TACGGCCAAATCCGTTCACA-3'

Note: RT-qPCR, reverse transcription quantitative polymerase chain reaction; F, forward; R, reverse; miR, microRNA; OCN, osteocalcin; PER2, period circadian protein 2; GAPDH, glyceraldehyde-3-phosphate dehydrogenase.
